# Population biology of *Ellochelon vaigiensis* (Quoy & Gaimard, 1825) in the Mekong Delta, Vietnam

**DOI:** 10.7717/peerj.14901

**Published:** 2023-02-21

**Authors:** Quang Minh Dinh, Ton Huu Duc Nguyen, Ngon Trong Truong, Tien Thi Kieu Nguyen

**Affiliations:** 1 Department of Biology, School of Education, Can Tho University, Can Tho, Vietnam; 2 Department of Molecular Biotechnology, Institute of Food and Biotechnology, Can Tho University, Can Tho, Vietnam; 3 Department of Biology, An Khanh High School, Can Tho, Vietnam

**Keywords:** *Ellochelon vaigiensis*, Exploitation, Length-frequency, Mortality, Mugilidae

## Abstract

*Ellochelon vaigiensis* is widely distributed and plays a vital role in the fisheries in the Mekong Delta (MD), but data on its population biological traits have not been known. Consequently, this study was conducted to provide data on its population biology used for fishing status assessment and fish resources management. Fish specimens were collected using trawl nets in two regions of the Hau River mouth, including northern in Ben Tre and Tra Vinh (BTTV) and southern in Soc Trang and Bac Lieu (STBL). Fish population biological parameters were estimated using FiSAT II software based on the fish length-frequency data. The male and female length-frequency data in each ecoregion were pooled together. Data analysis of 1,383 individual fish showed the sex ratio of the species was 1.00:1.30 at BTTV (309 females and 402 males) and 1.00:1.25 STBL (299 females and 373 males). Most fish specimens were caught at 12–22 cm total length (914 individuals), accounting for 66.09% of the fish collection. The difference in salinity between these two regions could affect the population biological parameters of *E. vaigiensis*. There were five cohorts (*e.g.*, five growth curves) in the BTTV and STBL. The von Bertalanffy curves of fish populations at BTTV and STBL were *L* = 33.6 (1 −*e*^−0.46(*t* + 0.34)^) *L* = 31.5 (1 −*e*^−0.56(*t* + 0.29)^), respectively. The growth index (Φ′) of this species at STBL (2.74) was higher than that at BTTV (2.72), whereas its longevity at BTTV (6.52 yrs) was higher than at STBL (5.36 yrs). The biomass and relative yield parameters, including *E*_0.1_, *E*_0.5_ and *E*_max_ were 0.358, 0.265 and 0.436 at BTTV; and 0.418, 0.293, and 0.513 at STBL, respectively. The fishing (*F*), natural (*M*), and total (*Z*) mortalities were 0.35/yr, 1.06/yr, and 1.41/yr at BTTV; and 0.55/yr, 1.24/yr, and 1.78/yr at STBL, respectively. The BTTV and STBL population was not overexploited because the exploitation rate (*E*_BTTV_ = 0.25, *E*_STBL_ = 0.31) was lower than *E*_0.1_ (0.358 in BTTV and 0.418 in STBL).

## Introduction

The Squaretail mullet *Ellochelon vaigiensis* (Quoy & Gaimard, 1825) is the monotypic species of the genus *Ellochelon* belonging to the family Mugilidae. This species is found from India to the Pacific Ocean (Crosetti & Blaber, 2015; Froese & Pauly, 2022). This fish is described with standard features such as 16 pectoral-fin rays; 25–29 longitudinal scales; 16 conjunctival scales; four hard dorsal fin rays, 9–10 soft dorsal-fin rays, three hard anal rays, 7–9 soft anal rays, pectoral fin black, caudal fin quite flat (Harrison & Senou, 1997; Tran et al., 2013). Most mullets are classified as saltwater fish because they often gather in schools and feed along coastal regions; however, *E. vaigiensis* can be found in brackish and freshwater areas about 10 km from the sea (Froese, 2006). This fish is distributed mainly in the surface layer with depths ranging from 0 to 5 m (Bacchet, Zysman & Lefèvre, 2006). *Ellochelon vaigiensis* belongs to the multiple spawning fish, and the fry, after hatching, can be found in mangroves (Breder & Rosen, 1966). Although *E. vaigiensis* plays a vital role in the food supply, especially in the estuarine and coastal region of the Mekong Delta (MD), its knowledge is limited to morphology, distribution (Penrith & Penrith, 1967; Kwun et al., 2013; Teimori & Hesni, 2020; Nguyen, Nguyen & Dinh, in press; Nguyen, Nguyen & Dinh, 2022), growth pattern and feeding habit (Dinh et al., 2022a; Dinh et al., 2022b). Besides, in the MD, especially downstream of Hau River—one of the tributes of the Mekong River, the survival and development of *E. vaigiensis* population face several reasons: exploitation pressure for food demand, pollution, and climate change (Tan & Thanh, 2013). Our preliminary observation shows that the number of fish individuals per catch tends to be decreasing trend, yet there have been no studies investigating this issue in the study area in order to verify whether this fish is being over-exploited or not. Therefore, this study was carried out to have a more general view of the impact of fishing on the population of this fish.

According to Al-Husaini et al. (2002), biological parameters of the fish population are helpful for fishery assessment. In addition, the population biological parameters help to understand the growth and mortality of the fish population (Amezcua, Soto-Avila & Green-Ruiz, 2006). Length-frequency data are input data for population biological parameters analysis (Tran, 2010). The basic principle of the length-frequency method is the frequency distribution of fish lengths in a given age group. The length frequency describes the growth and abundance of the population at different times (Pham & Tran, 2004). The fish growth and asymptotic length relationship regulate fish growth between locations (Pauly & Munro, 1984). The first capture length helps determine the maximum size the fish can reach (Pauly, 1987). Longevity provides information about the maximum lifespan of fish (Taylor, 1958). The growth rate and mortality show plasticity, changing according to location and species, the first capture length is common in fish and this is due to the trade-off between reproductive investment, growth, and mortality (Pauly, 1984).

Several studies on mullet populations have been carried out worldwide, *e.g.*, Mehanna (2004) details some parameters in *Liza carinata* and *Liza aurata* populations, such as the initial asymptotic length, growth parameter, longitude, and growth performance. These parameters have also been documented for *Liza saliens* in Beymelek Lagoon (Balik et al., 2011), *Rhinomugil corsula* in Bangladesh (Ara et al., 2019), and *Mugil cephalus* in India (Waters, 2014). However, population biological traits of *E. vaigiensis*, a target catching fish in the MD, has not been known. Moreover, the salinity gradually increased from the northern and southern Hau River mouth (Dinh et al., 2021), resulting in the variation of some biological parameters of two *Glossogobius sparsipapillus* populations between two regions (Nguyen et al., 2021). Therefore, this study aims to provide new data on the population parameters of *E. vaigiensis*, *e.g.*, first capture length, longevity, growth, exploitation rates, and mortalities. Moreover, the present study also presented the variations of these parameters between the northern and southern parts of the Hau River mouth. The findings will help to sustain this fish in MD.

## Materials & Methods

### Study sites and fish collection

**Figure 1:**
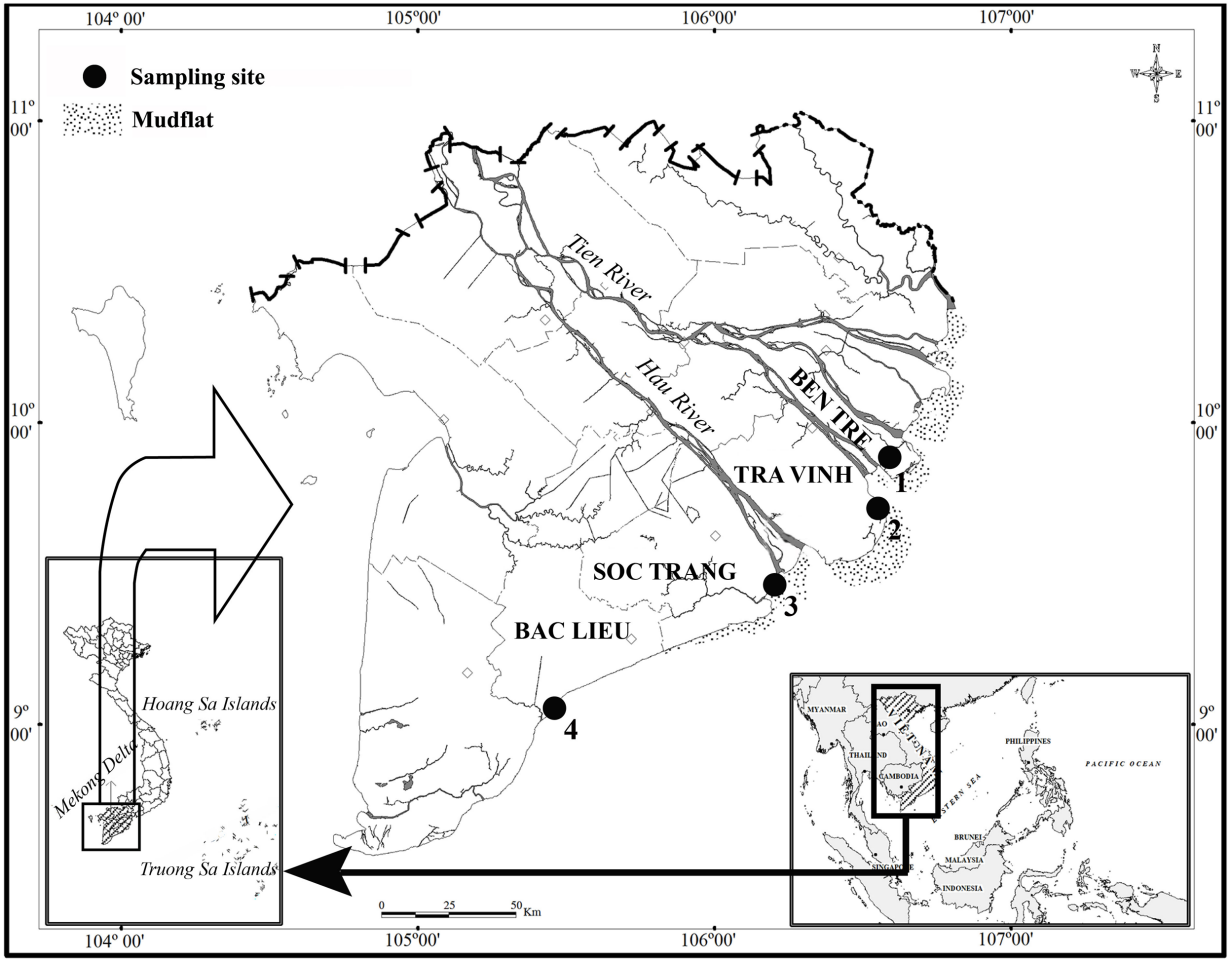
The sampling site (⋅) in the Mekong Delta modified from Dinh (2018). (1, Thanh Phu, Ben Tre; 2, Duyen Hai, Tra Vinh; 3, Tran De, Soc Trang; 4, Dong Hai, Bac Lieu).

This study lasted in two ecoregions: northern and southern Hau River mouth. Fish specimens were caught at two sites per ecoregion, including Ben Tre and Tra Vinh (BTTV) in the northern part and Soc Trang and Bac Lieu (STBL) in the southern part (Fig. 1). These two ecoregions were different salinity values, ranging from 12.9–13.7‰at BTTV to 16.9–29.6‰at STBL. At each site, fish specimens were caught monthly by trawl nets (mesh size of the codend: 2a = 1.5 cm) from November 2020 to March 2022. After collection, fish specimens were distinguished from other species by morphological features (Tran et al., 2013) before being anaesthetized with MS222 and transported to the laboratory. Fish specimens were then measured in the total length (*L*). Total length was determined from the mouth to the end of the fish’s tail. The use of fish in the present study was assessed and approved by the Scientific Committee of the School of Education, Can Tho University, under the Animal Welfare Assessment number BQ2020-05/KSP.

### Data analysis

Fish population biological parameters were estimated using FiSAT II software based on statistics on the fish length-frequency data (Gayanilo, Sparre & Pauly, 2005). The length-frequency data of males and females were pooled together because size by sex was insufficient for analysis. The stepwise procedure recorded by Amarasinghe & De Silva (1992) was applied to lessen length-frequency bias due to gear selection. Accordingly, the original length-frequency data in each region were used to calculate the initial asymptotic length (*L*_∞_) using the Powell-Wetherall procedure (Powell, 1979; Pauly, 1986b; Wetherall, 1986). Next, the *L*_∞_, according to Gayanilo, Sparre & Pauly (2005), was proposed to calculate the *K* index (growth parameter) from the ELEFAN I routine performed using the command Access/Direct fit of F/L data/ELEFAN I/K Scan/Compute. Then, both initial *L*_∞_ and *K* were used to get the probabilities of the capture file which was used to obtain the corrected length-frequency data from the trawl-type selection procedure by putting *L*_25_, *L*_50_, and *L*_75_ (Pauly, 1986a). Finally, the adjusted length-frequency statistics were applied to compute the final *K* and *L*_∞_ values using ELEFAN I (Gayanilo, Sparre & Pauly, 2005). These two values and the length-converted length were then used to calculate total mortality (*Z*) (Pauly, 1983). Meanwhile, the natural mortality (*M*) was determined as Log*M* = −0.007–0.279 Log *L*_∞_ + 0.654 Log*K* + 0.463 Log*T*, where *T* was the mean yearly water temperature, °C (Pauly, 1980). After obtaining, *Z* and *M* were used to estimate the fishing mortality (*F*) from the formula *F* = *Z*−*M*, and the exploitation rate (*E*) was calculated as *E* = *F*/*Z* (Ricker, 1975).

*Ellochelon vaigiensis* growth curves in each region were obtained using ELEFAN I from the final *L*_∞_ and *K* data (Pauly & David, 1981; Pauly, 1982; Pauly, 1987). The theoretical age parameter (*t*_0_) was determined through the equation Log_10_ (−*t*_0_) = −0.3922–0.2752 log_10_ *L*_∞_ − 1.038 log10*K* (Pauly, 1979). The *E*_max_ (maximum yield exploitation rate), *E*_0.1_ (optimal exploitation ratio), and *E*_0.5_ (exploitation rate with 50% stock reduction) were determined using the knife-edge method. Besides, the fishing status was estimated from the isopleth (*L*_*c*_/*L*_∞_) (Gayanilo, Sparre & Pauly, 2005). Since *L*_∞_ and *K* were not species-specific, Φ′ (*e.g.*, growth performance index) was utilized to collate the growth curves of this fish in the two ecoregions and with other species living in and out of MD. This coefficient was calculated using the formula Φ′ = Log*K* + 2Log*L*_∞_. The longevity (*t*_max_) was identified by the equation as *t*_max_ = 3/*K* (Taylor, 1958).

## Results

A total of 1,383 individuals (608 females and 775 males) were collected at BTTV and STBL. In both of these sampling sites, there were more males than females. At BTTV, the number of males and females was 402 and 309, respectively. Similarly, at STBL, this number was 299 for females and 373 for males.

**Figure 2:**
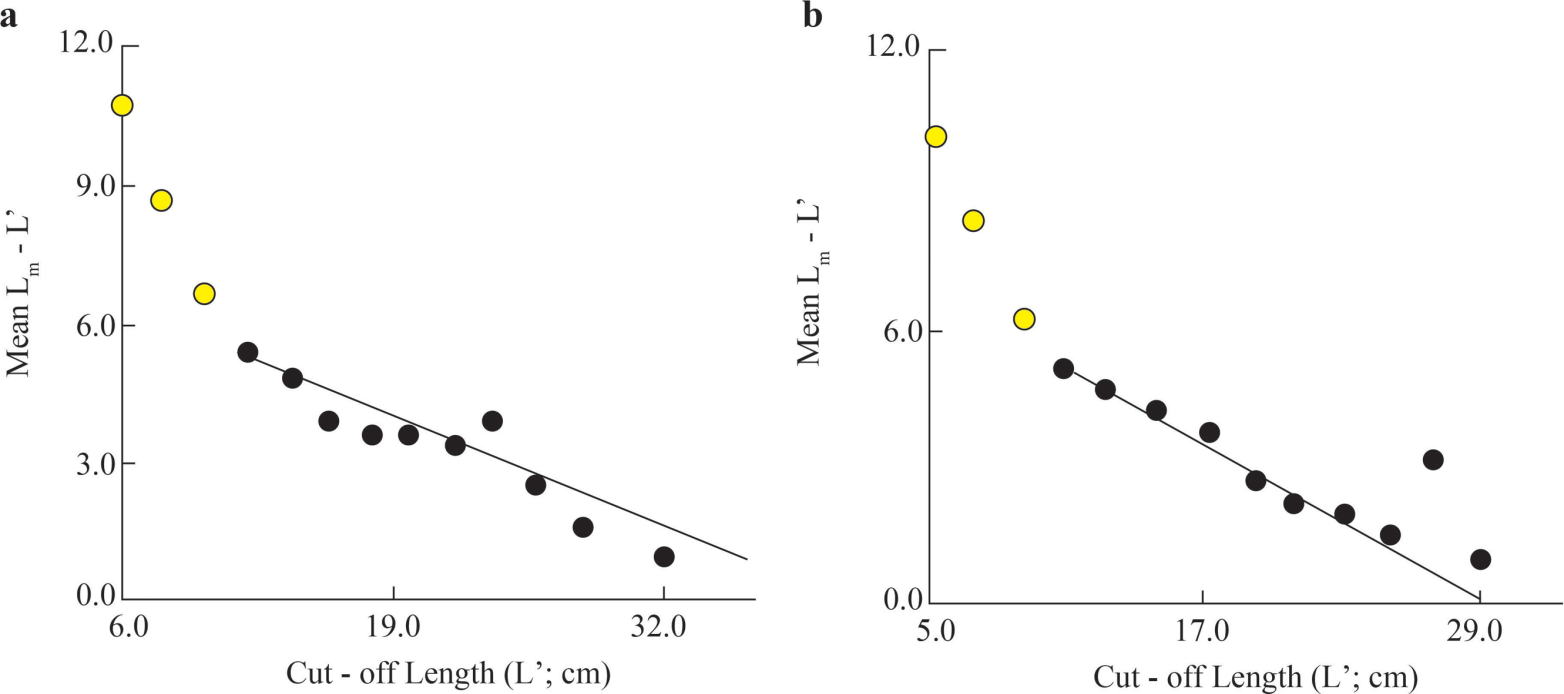
The *Ellochelon vaigiensis* Powell-Wetherall plot at BTTV (A) and STBL (B). BTTV, Thanh Phu, Ben Tre and Duyen Hai, Tra Vinh; STBL, Tran De, Soc Trang and Dong Hai, Bac Lieu.

**Figure 3:**
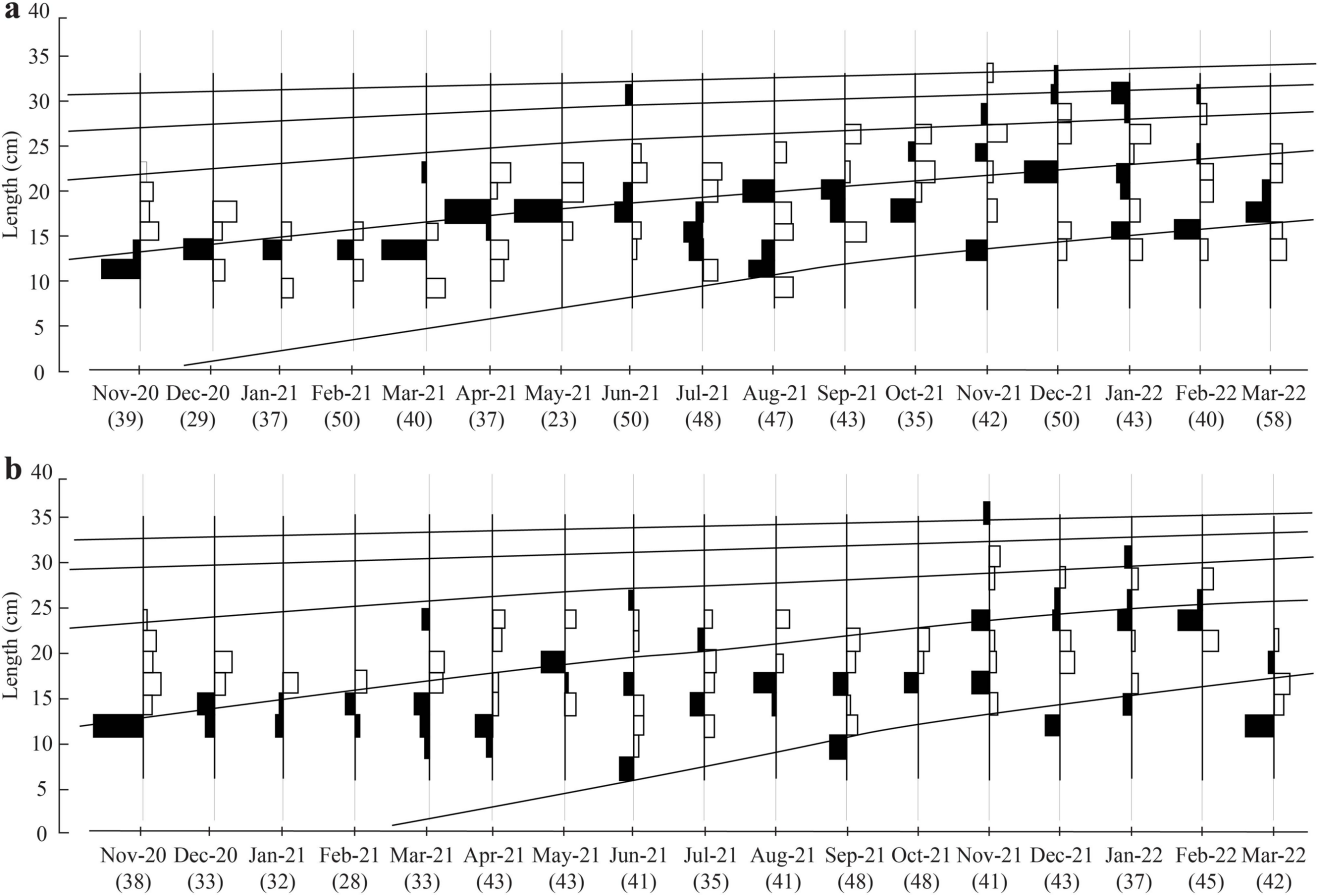
The *Ellochelon vaigiensis* growth curves at BTTV (a, *n* = 711) and STBL (b, *n* = 672). BTTV, Thanh Phu, Ben Tre and Duyen Hai, Tra Vinh; STBL, Tran De, Soc Trang and Dong Hai, Bac Lieu; number in brackish: individuals fish caught per month.

The initial *L*_∞_ of BTTV and STBL populations obtained from Powell-Wetherall plot was presented in Fig. 2. These graphs gave *L*_∞_ of the two populations (33.6 at BTTV and 31.5 at STBL) and *Z/K* (1.46 at BTTV and 2.12 at STBL). The length-frequency analysis (raw data can find: Raw data_*Ellochelon vaigiensis*) showed that the initial *K* was 0.46 at BTTV and 0.56 at STBL. The total length of *E. vaigiensis* at BTTV ranged from 6.3 to 32.6 cm *L*. The ELEFAN I analysis results showed that the fish population at BTTV had five distinct growth curves (Fig. 3A). Meanwhile, at STBL, the fish population had a total length ranging from 6.2 to 30.2 cm *L* and displayed five growth curves (Fig. 3B). The parameters of the growth curves were *L*_∞_ = 33.6 cm, *K* = 0.46/yr, *t*_0_ = −0.34 at BTTV and *L*_∞_ = 31.5 cm, *K* = 0.56/yr, *t*_0_ = −0.29 at STBL, respectively. The BTTV population exhibited the von Bertalanffy growth curve as *L* = 33.6(1 − *e*^−0.46(*t*+0.34)^), and this growth curve of the STBL population was *L* = 31.5(1 − *e*^−0.56(*t*+0.29)^).

**Figure 4:**
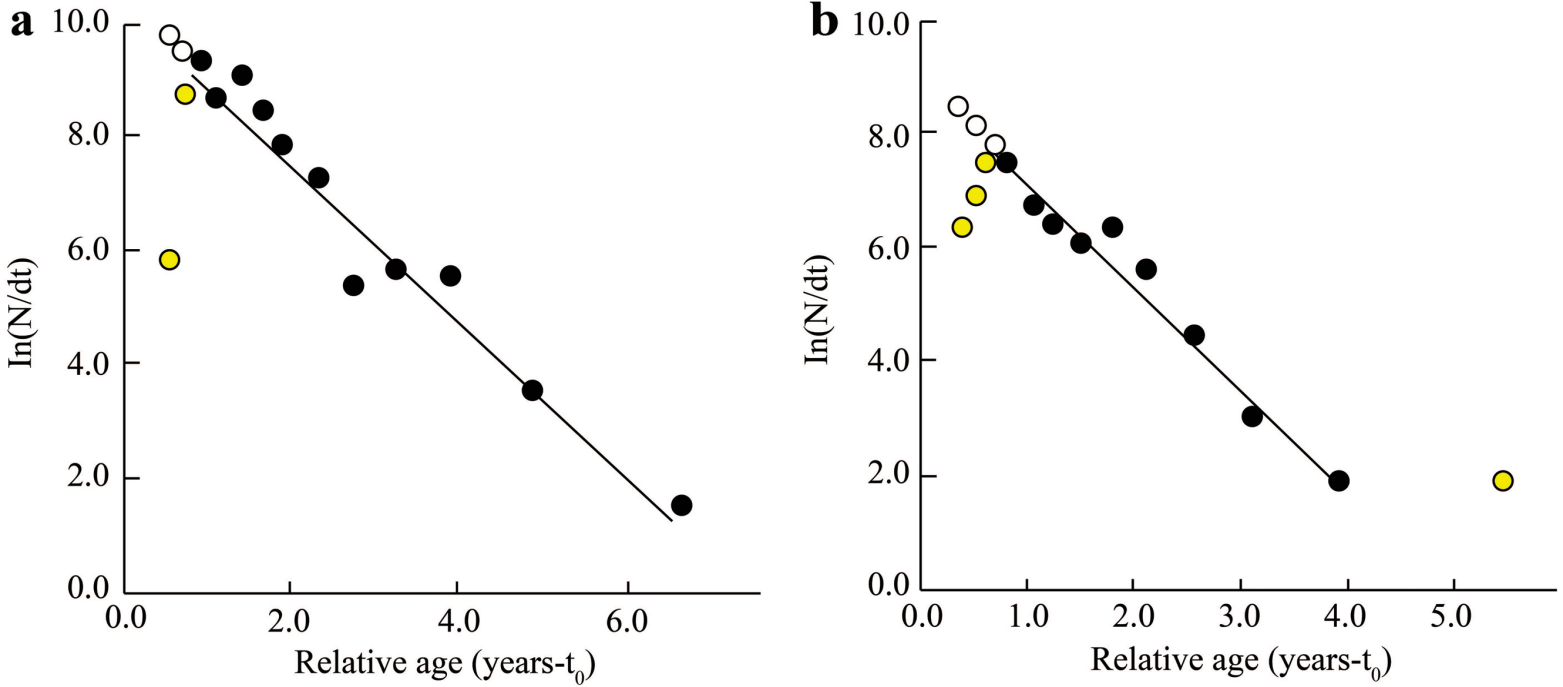
The *Ellochelon vaigiensis* length-converted catch curve. (A) Thanh Phu, Ben Tre and Duyen Hai, Tra Vinh, *Z* = 1.41 yr^−1^, *M* = 1.06 yr^−1^, *F* = 0.35 yr^−1^, *E* = 0.25; (B) Tran De, Soc Trang and Dong Hai, Bac Lieu, *Z* = 1.78 yr^−1^, *M* = 1.24 yr^−1^, *F* = 0.55 yr^−1^, *E* = 0.31.

The *Z*, *M*, *F*, and *E* determined from fish length-frequency data were 1.41/yr, 1.06/yr, 0.35/yr, and 0.25 at BTTV (Fig. 4A), and 1.78/yr, 1.24/yr, 0.55/yr and 0.31 at STBL (Fig. 4B), respectively. The study results also showed that the first length at first capture STBL (6.12 cm) was shorter than the BTTV (8.63 cm).

**Figure 5:**
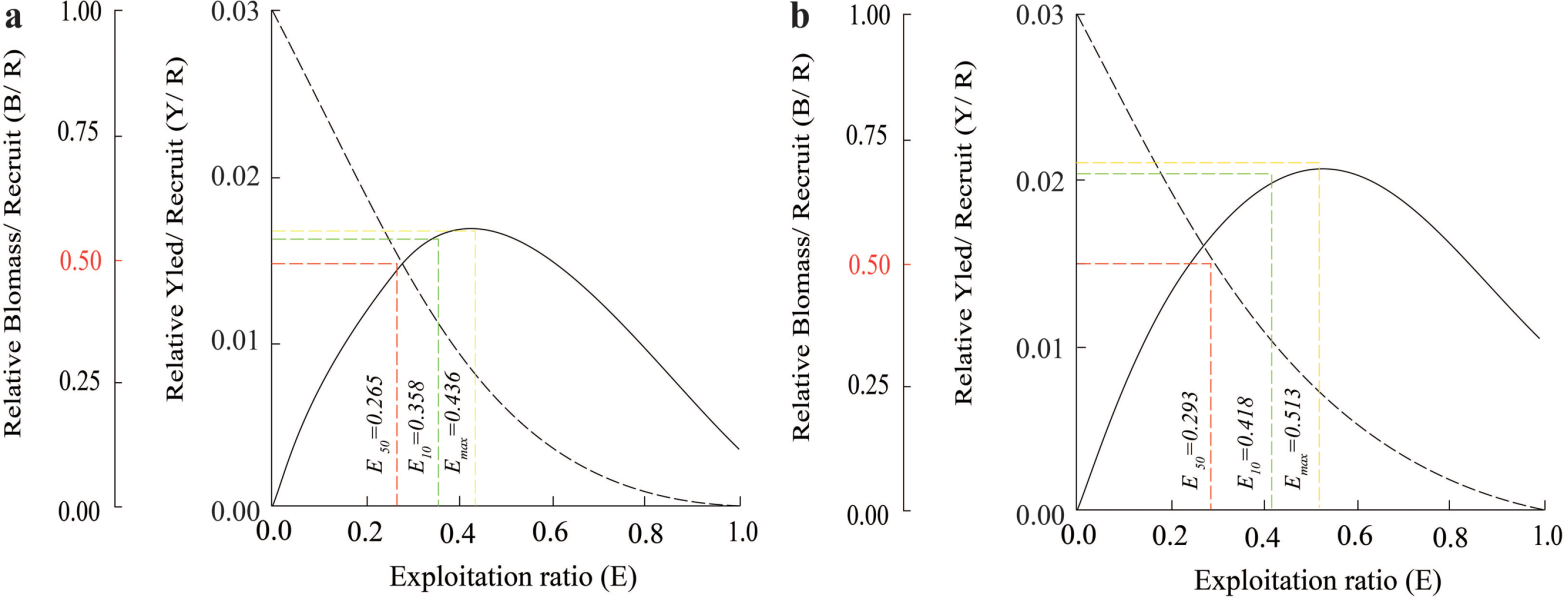
The *Ellochelon vaigiensis* relative biomass/recruit and relative yield/recruit. (A) Thanh Phu, Ben Tre and Duyen Hai, Tra Vinh; (B) Tran De, Soc Trang and Dong Hai, Bac Lieu.

**Figure 6:**
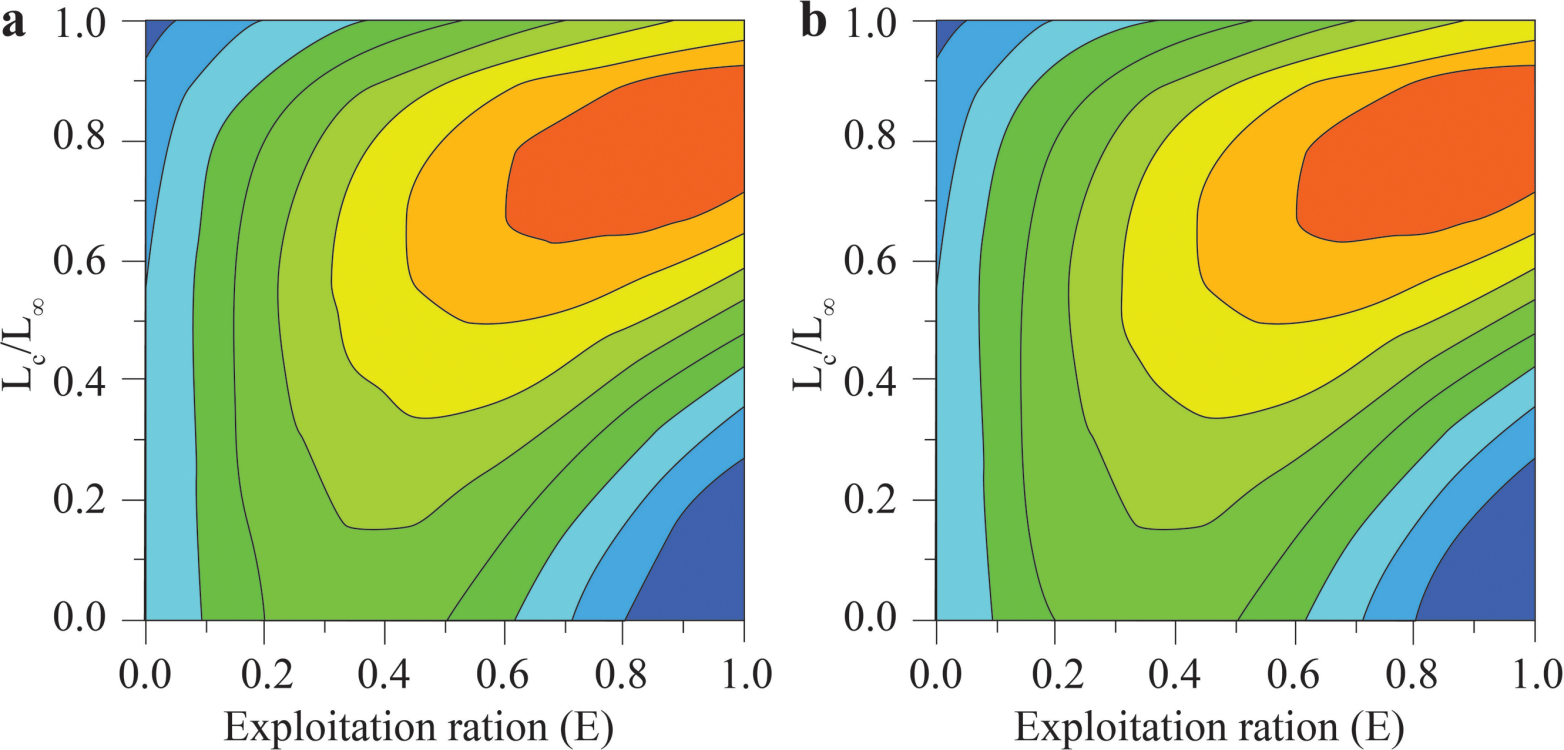
The *Ellochelon vaigiensis* relative yield isopleth diagram. (A) Thanh Phu, Ben Tre and Duyen Hai Tra Vinh, *L*_*c*_ = 6.12 cm, *L*_∞_ = 33.6 cm, *L*_*c*_/*L*_∞_ = 0.18; and (B) Tran De, Soc Trang and Dong Hai, Bac Lieu, *L*_*c*_ = 8.63 cm, *L*_∞_ = 31.5 cm, *L*_*c*_/*L*_∞_ = 0.27.

The *Y*′/*B* analysis result showed that the *E*_max_, *E*_0.1_, and *E*_0.5_ of the BTTV population were 0.436, 0.358, and 0.265, respectively (Fig. 5A). Meanwhile, the values of these parameters in the STBL population were 0.513, 0.418 and 0.293, respectively (Fig. 5B). The *t*_max_ of this fish was 6.52 yrs at BTTV and 5.36 yrs at STBL. Its growth performance index was 2.72 at BTTV and 2.74 at STBL (Table 1). The isopleth ratio (*L*_*c*_/*L*_∞_) of *E. vaigiensis* was 0.18 at BTTV (Fig. 6A) and 0.27 at STBL (Fig. 6B).

**Table 1:** Population parameters of *Ellochelon vaigiensis* and other mullets.

**Species**	***L*_∞_**	***K***	***t*_0_**	***t*_max_**	***Z***	***M***	***F***	***L*_*c*_**	***L*_*c*_/*L*_∞_**	***E***	**Φ′**	**Distribution**	**Reference**
*Liza abu*	25.4	0.24	−1.57	12.5	–	–	–	–	–	–	–	Iraq	Tulkani (2017)
*Chelon labrosus*	83.9	0.08	−0.79	35.5	–	–	–	–	–	–	2.75	North West Wales	Tulkani (2017)
*Liza argentea*	29.72	0.33	−0.89	9.09	0.61	0.37	0.24–0.32	–	–	0.39–0.52	–	Australia	Kendall, Gray & Bucher (2009)
*Myxus elongatus*	35.47	0.48	−0.13	6.25	0.92	0.40	0.43–0.52	–	–	0.47–0.57	–		Kendall, Gray & Bucher (2009)
*Planiliza abu*	21.2	0.44	−0.42	6.82	2.52	1.08	1.44	9.40	0.44		2.30	Iraq	Mohamed & Abood (2020)
*Planiliza klunzengeri*	27.0	0.49	−0.23	6.12	3.16	1.09	2.07	13.97	0.52	–	2.62	Iraq	Mohamed & Abood (2020)
*Planiliza subviridis*	29.3	0.40	−0.42	7.50	1.68	0.93	0.75	12.84	0.44	–	2.54	Iraq	Mohamed & Abood (2020)
*Eleochelon vaigiensis*	35.70	0.68	−0.24	4.41	2.05	1.34	0.71	14.01	0.39	0.34	2.93	BTTV	This study
	32.40	1.40	−0.11	2.14	4.80	2.23	2.57	9.72	0.30	0.54	3.17	STBL	This study

**Notes.**

*L*_∞_ the asymptotic length *K* growth parameter *t*_0_ theoretical age parameter *t*_max_ longevity *Z* total mortality *M* natural mortality *F* fishing mortality *L*_*c*_ Length at first capture *L*_*c*_/*L*_∞_ isopleth E exploitation ratio Φ′ growth performance BTTV Thanh Phu, Ben Tre and Duyen Hai, Tra Vinh STBL Tran De, Soc Trang and Dong Hai, Bac Lieu

## Discussion

The maximum length of *E. vaigiensis* tended to vary with the salinity changes, as it reached 31.5 cm at STBL with a salinity of 12.9 to 13.7‰and 33.6 cm at BTTV with a salinity of 16.9–29.6‰in the present study. Indeed, according to Nikolsky (1963), differences in environmental conditions such as water quality, food sources, and extraction processes can affect the maximum length of fish. In the previous study, this species reached the maximum length of 63 cm in the Red Sea (Lieske & Myers, 1994), where salinity ranged from 36 to 40‰(Sofianos & Johns, 2003), suggesting that this fish could adapt well to higher salinity regions. This change is not only recorded in mullet but also in some other fish species. For example, the maximum length of *G. sparsipapillus* was inversely proportional to salinity as *L*_∞_ in *G. sparsipapillus* is lower than in high salinity region than in low salinity one (Nguyen et al., 2021), seeming that the *L*_∞_ tended to change differently for these species when changing salinity. On the other hand, in *Periophthalmodon septemradiatus*, *L*_∞_ remained unchanged between freshwater and brackish water regions (Tran & Dinh, 2020). The maximum size of *E. vaigiensis* was relatively higher than some other mullets. For example, according to Mohamed & Abood (2020), in Iraq, the size of *Planiliza abu* (6.4 to 19.7 cm), *P. klunzengeri* (6.0–19.0 cm), *P. subviridis* (9.8–26.5 cm) was smaller in *E. vaigiensis*. Meanwhile, some other species of mullet, which were widely distributed in Australia but had a longer *L*_∞_ than *E. vaigiensis,* such as *Liza argentea* (5.9–34.6 cm) and *Myxus elongatus* (5.7–39.3 cm) (Kendall, Gray & Bucher, 2009). Variations in *K* at each location affect *L*_∞_ and longevity, as there was an inverse relationship between *L*_∞_ and *K*. At BTTV, the *L*_∞_ was higher, but the *K* was lower; thus, the lower growth rate leads to a higher *L*_∞_, with the same number of curves, but with a higher *t*_max_ than that observed for STBL. The values between the locations were close, but their variation consequently generated variations with related parameters, such as higher *K*, lower *L*_∞_, and lower longevity. The plasticity of *K* between locations for the same species was evident. These results showed that salinity may regulate the change of *K*, *L*_∞_ , and *t*_max_ of *E. vaigiensis* and other mullets. Besides salinity, other biotic and abiotic factors could also influence this change, but these factors in the two ecoregions have not been studied yet, suggesting that there is a need to clarify if other environmental factors influence the variation of these population paremeters of *E. vaigiensis* and other mullets.

The Φ′ at BTTV (2.72) was lower than in the STBL(2.74), showing that the southern region of Hau River mouth was suitable for developing this species, or this fish tended to grow well in the higher salinity areas. However, the difference between these two regions was not too great. The Φ′ of this species was higher than that of some other mullets. Indeed, Φ′ of males, females, and whole populations of *L. abu* in the Euphrates River, AlNajaf, Iraq, were 2.27, 2.29, and 2.23, respectively (Tulkani, 2017), showing that *L. abu* displayed a lower growth rate than *E. vaigiensis*. The other three fish species, including *P. abu*, *P. klunzengeri*, *P. subviridis* in Al-Arab River, Iraq, exhibited different growth rates of 2.30, 2.62, and 2.54, respectively (Mohamed & Abood, 2020), and these values were generally smaller than *E. vaigiensis* (Table 1). At the same time, *Chelon labrosus* in North West Wales (Tulkani, 2017) and *M. elongatus* in Australia (Kendall, Gray & Bucher, 2009) displayed a relatively high growth rate with values of 2.75 and 2.78, respectively (Table 1). These results showed that although the same group of mullets, each species exhibited a different growth rate. This difference may be due to the fact that this was characteristic of each species. However, from the above examples, it can be seen that marine mullet species such as *E. vaigiensis* in MD, *C. labrosus* in North West Wales, and *M. elongatus* in Australia have relatively larger growth rates compared to other species. species distributed in river basins in Iraq. Thereby, it can be seen that the growth rate of fish depends not only on the characteristics of the species but also on the ecological conditions, habitat, food source, metabolic activity, reproductive activity, size of fish, sampling method, and fishing pressure in each area (Nikolsky, 1963; Panda et al., 2018).

The longevity (*t*_max_) of *E. vaigiensis* varied with ecoregion, as this value at STBL was lower than at BTTV (Table 1). The possible reason is that variation in salinity between these regions regulated *t*_max_. The overexploitation at STBL could also lead to a lower *t*_max_ in the STBL population than in the BTTV population. Besides, the fish population at STBL grew faster than BTTV, leading to faster fish maturation and the end of the life cycle. The spatial variations in *t*_max_ and Φ′ were also found in *L. argentea* and *M. elongatus* distributed in Australian estuaries (Kendall, Gray & Bucher, 2009). Compared to other mullets, *E. vaigiensis* exhibited shorter longevity. Kendall, Gray & Bucher (2009) reported that *L. argentea* and *M. elongatus* showed a longer *t*_max_, reaching 9.09 yrs and 6.25 yrs, respectively. According to these authors, these two fish displayed a long lifespan due to being less affected by human exploitation. The *t*_max_ of three other mullets, *e.g.*, *P. abu*, *P. klunzengeri,* and *P. subviridis* in Al-Arab River, Iraq, was 6.82, 6.12, and 7.50 yrs, respectively (Mohamed & Abood, 2020). Meanwhile, the *t*_max_ of *L. abu* in Central Iraq and *C*. *labrosus* in Northwest Wales reached 12.5 and 35.5 yrs, respectively (Tulkani, 2017) (Table 1). From these results, it is shown that the life expectancy of fish is not only affected by environmental conditions but also by human activities.

Total mortality (*M*) and natural mortality (*Z*) of the STBL population were higher than those in the BTTV population, suggesting that the environmental condition at STBL was less favorable than at BTTV. Fishing gears were increasingly developed in extermination (use electricity, small mesh), leading to higher mortality in this species. So, it is necessary to re-regulate the appropriate fishing gear when exploiting this species to protect them from being overexploited. Compared with two Australian mullets, *e.g.*, *L. argentea* and *M. elongatus* (Kendall, Gray & Bucher, 2009), *M* and *Z* of *E. vaigiensis* were higher. However, in three fish species in Iraq (Tulkani, 2017), *M* was higher than those of the BTTV population but lower than those of the STBL. Meanwhile, the *Z* value of these three fish species was almost higher than that of *E. vaigiensis* (Table 1). These three fish in Irad seemed more vulnerable to exploitation and responsible for higher mortality. It could be because fishes in Iraq were caught in the riverine region (freshwater), whereas *E. vaigiensis* in the present study was caught in the estuarine one (brackish water). Besides, environmental conditions in MD were relatively stable between seasons compared to Australia and Iraq, leading to a lower natural mortality rate of this fish than some other fish species.

Both populations in BTTV and STBL have not been overexploited as *E* was <*E*_0.1_. It showed that although this fish had high economic value and was intensely exploited, the fish population still met the fishing demand. Thus, the current exploitation intensity and mesh size were suitable for developing *E. vaigiensis*. Depending on the method used to estimate *M*, the estimate of E may be slightly smaller or larger (Pauly, 1980). But the frequency of this fish in two ecoregions showed the occurrence of many groups of fish of mature size. However, measures should be taken, such as limiting the fishing of these fish during the spawning period, to manage this resource sustainably in the MD. This activity could avoid overexploited as in some fish species in the MD, *e.g.*, *G*. *giuris* (Dinh, Phan & Tran, 2017), *G*. *aureus* (Dinh, Tran & Tran, 2021), and *P. schlosseri* (Tran & Dinh, 2021).

The *L*_*c*_/*L*_∞_ of *E. vaigiensis* (0.18 at BTTV and 0.27 at STBL) were lower than that of other mullets, *e.g.*, *P. abu* (0.44), *P. subviridis* (0.44) and *P. klunzengeri* (0.52) in Iraq (Tulkani, 2017) (Table 1). This suggested that in MD, *E. vaigiensis* was caught at an earlier stage than other mullets in Iraq. Compared to some fish species belonging to other families living in MD, *E. vaigiensis* was found to be caught in an earlier stage as its *L*_*c*_/*L*_∞_ was lower than that of them, *e.g.*, *Parapocryptes serperaster* (0.57) (Dinh, Qin & Tran, 2015), *Boleophthalmus boddarti* (0.77) (Dinh, 2017), and *Trypauchen vagina* (0.57) (Dinh, 2018) (Table 1). This could be caused by the higher economic values of *E. vaigiensis* than these fish species in MD. On the other hand, the distribution area of this fish was mainly the estuary, and along the coast, so the fish is easily exploited (McDowall, 1997). Meanwhile, *Parapocryptes serperaster* or *Boleophthalmus boddarti* were mainly distributed in the alluvial zone, so it was more difficult to exploit than the species in this study. To conserve and sustain the fish resources, the local government should ask fishers to increase the size at first capture >*L*_*m*_ (19.61 cm at BTTV and 18.50 cm at STBL), which was the length in which 50% of fish reached maturity firstly and obtained from the equation Log_10_ *L*_*m*_ = 0.898 × log_10_(*L*_∞_) − 0.0782 (Froese & Binohlan, 2000).

The results of this study show that the biological parameters of fish populations may be regulated by environmental conditions in two ecoregions, particularly salinity. The fish size limitation of 6.3–32.6 cm at BTTV and 6.2–30.2 cm at STBL could be a bias influencing the variation of biological parameters of this fish population. Moreover, other environmental conditions of these two ecoregions, *e.g.*, flora and flow rate, could potentially affect the population’s biological parameters but have not yet been investigated. Therefore, there is a need to study the population relationship of this fish with other biological factors and try to expand the fish size range from <6.2 cm to >32.6 cm to take appropriate measures to protect and develop this resource in the future.

## Conclusion

This study provided data on the population biology of *E. vaigiensis* in two ecoregions, BTTV and STBL. This fish population grows better in areas with low salinity. The maximum length and longevity of *E. vaigiensis* were higher in the BTTV, but the growth performance index was lower in this area than in the STBL. The mortalities of this species at STBL were higher than those at BTTV. The STBL fish population is not overexploited. However, we need to suggest that locals should avoid catching fish with a length <*L*_50_ in order to conserve and sustain the fish resources.

##  Supplemental Information

10.7717/peerj.14901/supp-1 Data S1 Raw data: Ellochelon vaigiensis

## Additional Information and Declarations

### Field Study Permissions

The following information was supplied relating to field study approvals (*i.e.*, approving body and any reference numbers): In terms of the field trip, our research has no field permit requirement as we collect fish from the public-owned where local fishers collect fish daily. Moreover, we collect fish just once a month at each site, and the number of fish collection is within allowable limits of the animal assessment.

### Data Availability

The following information was supplied regarding data availability: The raw data is available in the Supplemental File.
